# Engineering an αCD206-synNotch Receptor: Insights into the Development of Novel Synthetic Receptors

**DOI:** 10.1021/acssynbio.4c00149

**Published:** 2024-11-18

**Authors:** Sofija Semeniuk, Bin-Zhi Qian, Elise Cachat

**Affiliations:** † Centre for Engineering Biology, University of Edinburgh, Edinburgh EH9 3BF, United Kingdom; ‡ Institute of Quantitative Biology, Biochemistry and Biotechnology, School of Biological Sciences, University of Edinburgh, Edinburgh EH9 3BF, United Kingdom; § Fudan University Shanghai Cancer Center; Department of Oncology, Shanghai Medical College, The Human Phenome Institute, Zhangjiang-Fudan International Innovation Center, Fudan University, Shanghai 200433, China

**Keywords:** synthetic receptor, synNotch, CD206, macrophages, metastasis, cross-reactivity

## Abstract

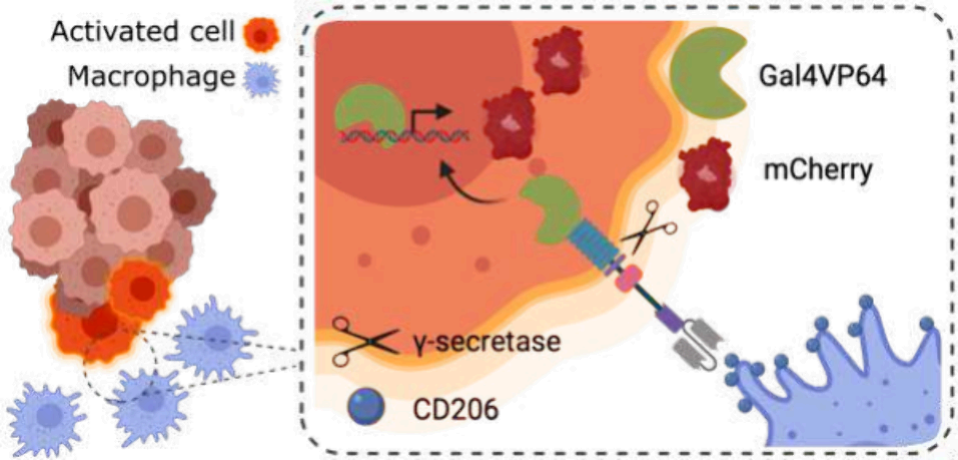

Immune cells play a pivotal role in the establishment, growth, and progression of tumors at primary and metastatic sites. Macrophages, in particular, play a critical role in suppressing immune responses and promoting an anti-inflammatory environment through both direct and indirect cell–cell interactions. However, our understanding of the mechanisms underlying such interactions is limited due to a lack of reliable tools for studying transient interactions between cancer cells and macrophages within the tumor microenvironment. Recent advances in mammalian synthetic biology have introduced a wide range of synthetic receptors that have been used in diverse biosensing applications. One such synthetic receptor is the synNotch receptor, which can be tailored to sense specific ligands displayed on the surface of target cells. With this study, we aimed at developing a novel αCD206-synNotch receptor, targeting CD206^+^ macrophages, a population of macrophages that play a crucial role in promoting metastatic seeding and persistent growth. Engineered in cancer cells and used in mouse metastasis models, such a tool could help monitor—and provide an understanding of—the effects that cell–cell interactions between macrophages and cancer cells have on metastasis establishment. Here, we report the development of cancer landing-pad cells for versatile applications and the engineering of αCD206-synNotch cancer cells in particular. We report the measurement of their activity and specificity, and discuss unexpected caveats regarding their *in vivo* applications.

## Introduction

The intercellular interactions, both direct and indirect, between malignant and immune cells play a significant role in cancer growth and progression.^1,2^ During all stages of cancer development through to metastasis formation, multiple subsets of immune cells can be found in the tumor microenvironment, such as cytotoxic cells (e.g., CD8^+^ T cells or NK cells), immunoregulatory Treg, Breg and T helper cells, as well as macrophages.^1^ These immune cell populations contribute to cancer cell establishment and tumor propagation through direct cell–cell contact or through indirect interaction via soluble cytokines.^1,3^ One of the most prominent immune cell types participating in these interactions are macrophages.^2,4−6^ In the tumor microenvironment (TME), monocytes are polarized toward either a pro-inflammatory or a pro-tumorigenic state, making them an important player in tumor development and progression.^4,5,7,8^

**Figure 1:**
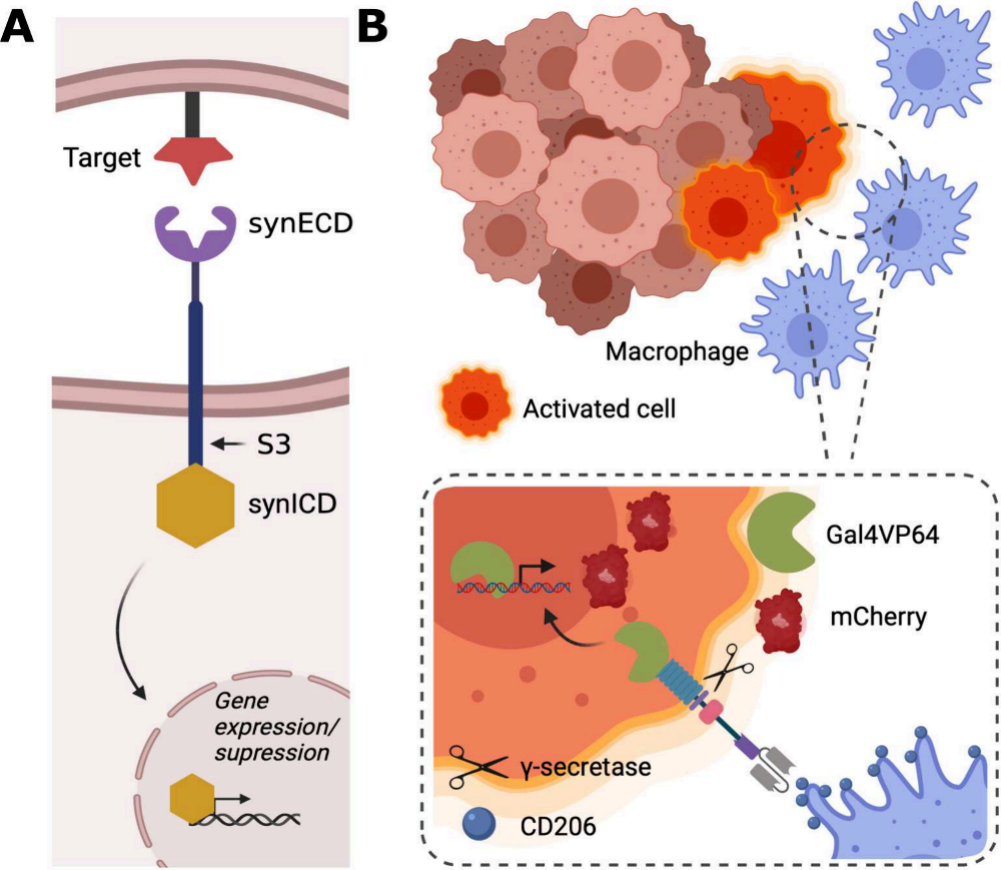
**The architecture and mechanism of synNotch receptors.** (A) The synNotch receptor consists of three modular domains: extracellular domain (synECD), notch core domain and intracellular domain (synICD). S3 indicates a crucial cleavage site, which is targeted by γ-secretase. Upon ligand recognition by the synECD, mechanical forces open S3, which leads to the release of the synICD. Translocation of synICD into the nucleus can be engineered to induce changes in expression of downstream genes of interest. (B) Schematic representation of the engineered macrophage-specific synNotch system. Cancer cells engineered with the macrophage-specific synNotch detect macrophages in the tumor microenvironment. Binding between the macrophage surface marker (in this case CD206) leads to the release of transcriptional activator Gal4VP64, which translocates to the nucleus and induces the expression of a reporter gene (mCherry). *Created with*BioRender.com.

Importantly, studying the processes that underlie immune cell reprogramming and cancer growth is challenging due to the transient nature of these interactions. Recent developments in synthetic biology offer receptor-based tools, which allow studying various biological processes such as tissue development^9,10^ and cell signaling.^11^ The use of synthetic receptors, derived from endogenous receptors but engineered to either detect novel ligands, elicit custom responses, or both, has been largely demonstrated in the published literature (reviewed elsewhere).^11^ In the context of this study, one such tool is the synthetic Notch (synNotch) receptor, which uses synthetic input and output modules and is one of the few receptors that specifically detect membrane-tethered ligands (Figure 1A).^10^ Multiple studies have already demonstrated the potential applications of synNotch in therapeutics and diagnostics,^10,12−20^ tissue morphogenesis,^9^ and fundamental studies.^21^

We aimed to develop a synNotch-based receptor-reporter system to monitor the transient interactions between cancer cells and immune cells both *in vitro* and *in vivo* in a mouse model of metastasis (Figure 1B). Unlike other synNotch research where the focus is engineering immune cells to target cancer cells, this study aims to engineer cancer cells with a macrophage-sensitive synNotch receptor targeting CD206, a surface marker specific to pro-tumorigenic macrophage subsets. Upon ligand recognition, the induction of a genetically encoded reporter results in a fluorescent response in engineered cancer cells. Extracting and sorting tumor cells into fluorescent (positive for macrophage contact) and nonfluorescent (negative for macrophage contact) cell populations can help decipher the pro-metastatic effects the cell–cell interactions between tumor and immune cells have on cancer cells and their survival, and could lead to the identification of new drug targets that can disrupt these effects. Here, we present the development of an anti-CD206 (αCD206)-synNotch receptor, together with the insights we gained through this study regarding receptor activity and specificity.

## Methods

### Molecular Biology

The αCD206-synNotch is comprised of an IgK leader peptide (derived from the Bornean orangutan T-cell surface glycoprotein CD8 alpha chain; MALPVTALLLPLALLLHAARP), myc tag, an αCD206 VHH sequence,^22^ Notch core domain and Gal4VP64 transcriptional activator.^10^ The αCD19-synNotch sequence was identical to the one published by Morsut et al.^10^ Both receptors were expressed under a mammalian phosphoglycerate kinase (PGK) promoter and had a bovine growth hormone polyadenylation (BGH polyA) sequence at the C terminal. The whole cassette was flanked by PiggyBac inverted terminal repeats (ITRs).

For the generation of MetBo2-CD206^+^, MetBo2-F4/80^+^ and MetBo2-CD19^+^ sender cells, CD206, F4/80 and CD19-expressing vectors were generated. The CD206 expression cassette consisted of a putative CD206 extracellular domain sequence (NM008625.2, 81–3835 nt), a myc tag, and a PDGFRβ transmembrane domain (derived from the transmembrane domain of the human platelet-derived growth factor receptor; AVGQDTQEVIVVPHSLPFKVVVISAILALVVLTIISLIILIMLWQKKPR). The CD206 sequence was extracted from IL-4 treated Bone Marrow Derived Macrophages (BMDMs). The F4/80 cassette consisted of an F4/80 coding sequence (NM010130.4, 21–2836 nt). The CD19 expression cassette was identical to the one published by Morsut et al.^10^ Both CD206 and CD19 cassettes were expressed under a cytomegalovirus (CMV) promoter and had a bovine growth hormone polyadenylation (BGH polyA) sequence at the C terminal.

All constructs and most essential primers used in this research are summarized in Tables S1 and S2, respectively.

### Cell Culture

#### Cell Lines

MetBo2 (polyoma middle T oncogene-induced mouse mammary tumor on a syngeneic Friend Virus B NIH Jackson (FVB) background)^23^ cells were maintained in 1X Dulbecco’s Modified Eagle Medium (DMEM) (Thermofisher Scientific; Cat. No. 11995065) with 10% Fetal Bovine Serum (FBS) (Sigma-Aldrich; Cat. No. F2442) and 1% Pen/Strep (Thermofisher Scientific; Cat. No. 15140122) or Antibiotic- Antimycotic (Thermofisher Scientific; Cat. No. 15240096). Cell cultures were kept at 37 °C with 5% CO_2_.

#### Transfections

Cells were seeded in 48- or 24-well plates 24 h prior to transfection. For transfections, Lipofectamine 3000 (Thermofisher Scientific; cat. no. L3000001) was used.

#### Co-cultures

For co-cultures, receptor and sender cells were mixed together at a 1:1 ratio and seeded in a cell culture plate. For a 24-well plate format, 0.5 × 10^5^ cells of each cell type was used. For a 48-well format, 0.3 × 10^5^ of each cell type was used. Cells were grown in the 37 °C incubator for 24 h prior to imaging or flow cytometry.

#### Flow Cytometry

For the acquisition of heterogeneous and monoclonal cell populations, cells were harvested 1X Accutase (Thermofisher Scientific; Cat. No. A1110501) and centrifuged at 1000 rpm for 5 min. The pellet was resuspended in 1 mL of sorting buffer (1X DPBS, 1% FBS, 10% penicillin/streptomycin) and centrifuged at 500 rpm for 5 min. The pellet was resuspended again in 0.5 mL of sorting buffer and kept on ice until the sorting. FACS sorting was carried out using a BD FACS Aria IIIu 4-laser/11 detector Cell Sorter (The University of Edinburgh Institute of Immunology & Infection Research Flow Cytometry Core Facility). Sorted cells were seeded in a recovery medium (1X DMEM, 20% FBS, 5% penicillin/streptomycin).

The flow cytometry experiments were carried out using BD Fortessa with FITC, PE, PE-Dazzle, PE-Cy5, PE-CY5.5, PE Texas Red, AlexaFluor700 and BV421 filters. Cells were washed using 1X DPBS and incubated for 5 min at 37 °C with 1X Accutase (Thermofisher Scientific; Cat. No. A1110501). Cells were harvested using flow buffer (1X DPBS, 1% FBS) and transferred to a 96-well plate for flow cytometry analysis.

In flow cytometry analysis, cells were first gated by size using forward and side scatters (SSC-A against FSC-A), and singlets were gated using forward scatters (FSC-A against FSC-W). Further gating was dependent on the type of experiment.

### Development of the MetBo2-UAS Cell Line

For the assessment of MetBo2-UAS clones, the mCherry fluorescence was analyzed directly following singled gating. MFI of mCherry was multiplied by the percentage of mCherry^+^ cells from the parent population (singlets) to get the total fluorescence of the cell population.

### Development and Analysis of the synNotch Cell Lines

Four days following transfection of MetBo2-UAS cells with the receptor cassette using the PiggyBac system, an initial FACS bulk sorting was carried out in order to enrich the population for BFP^+^ cells. This heterogeneous population was expanded and co-cultured with CD206^+^ sender cells at a 1:1 ratio. By default and unless otherwise stated, the sender cells were MetBo2 cells, transiently transfected with 250 ng of the CD206 expression plasmid in a 48 well plate. The second round of FACS single cell sorting was carried out 24 h post co-culture in order to isolate clones that exhibited elevated levels of mCherry fluorescence. The cells were gated by BFP fluorescence, therefore isolating only receptor cells. Subsequently, the MFI of mCherry in the BFP^+^ population was multiplied by the percentage of mCherry^+^ cells in the parent population of (BFP^+^) cells to get the total fluorescence of the cell population. Following the expansion of monoclonal αCD206-synNotch cell populations, each of them was presented to CD206^+^ sender cells in a 1:1 ratio. Receptor activation levels, indicated by elevated mCherry fluorescence, were assessed using flow cytometry through identical gating and analysis pipeline, and the clone which exhibited the highest signal-to-noise ratio was selected for further experiments.

All flow cytometry data analysis was carried out in FlowJo and GraphPad Prism.

### Immunostaining of C57BL/2 Mouse Spleen Extract

Mouse spleen extract, prestained with immune-cell specific antibodies, was acquired from the Binzhi Qian lab at the MRC Centre for Reproductive Health at The University of Edinburgh. The following antibodies were used: Alexa Fluor 700 anti-mouse F4/80 Antibody (Biolegend; Cat. No. 123129), PE anti-mouse CD206 (MMR) Antibody (Biolegend; Cat. No. 141705), PE/Cyanine5 anti-mouse/human CD45R/B220 Antibody (Biolegend; Cat. No. 103209), PE/DazzleTM 594 anti-mouse CD3 Antibody (Biolegend; Cat. No. 100245), PerCP/Cyanine5.5 anti-mouse/human CD11b Antibody (Biolegend; Cat. No. 101227). The extract was split into equal parts, and 250 to 500 μL of supernatant, containing the small antibody domain chromobodies, was loaded on the extract and incubated in the dark for 1 h at 4 °C. Next, the cells were washed twice with DPBS and analyzed using flow cytometry. Compensation was carried out using UltraComp eBeads Compensation Beads (Invitrogen; Cat. No. 01-2222-42).

### Chromobody Staining

Chromobodies were generated by transiently expressing the chromobody expressing plasmids in HEK293FT cells in a 6 well plate. The media was collected from the cells 2 days later and centrifuged to pellet the cells and cell debris. The supernatant was used to stain the fixed cells at 4 °C overnight in the dark.

### Fluorescent Microscopy

Fluorescent imaging was carried out using a Leica DMi8 fluorescent microscope with DAPI (Ex: 350/50, Em: 460/50), TexasRed (Ex: 560/40, Em: 630/75), GFP (Ex: 470/40, Em: 525/50) and Y5 (Ex: 620/60, Em: 700/75) filter cubes. Further image processing was carried out with FIJI software.

## Results

### Development of a Stable αCD206 synNotch Cell Line

**Figure 2:**
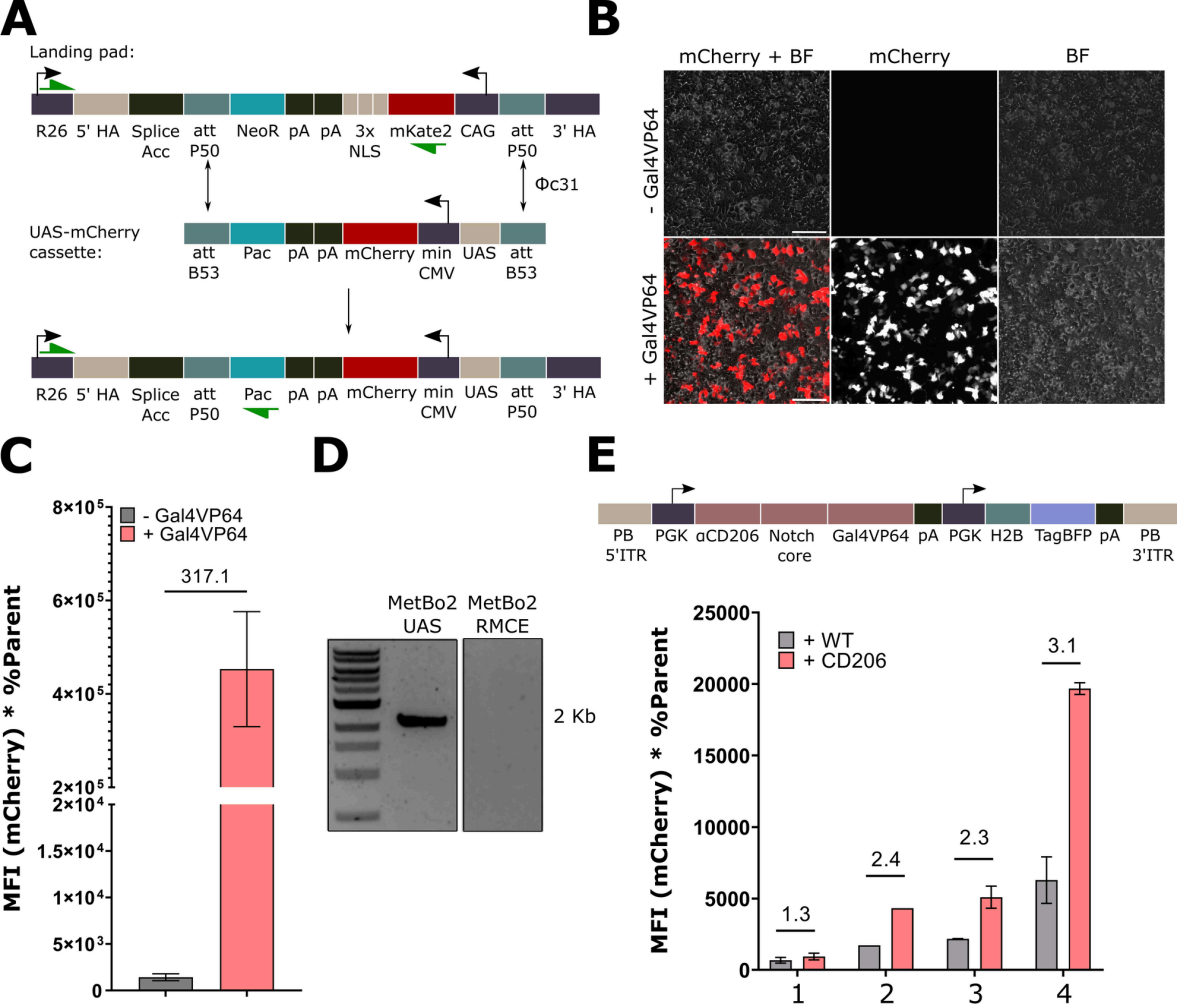
**Engineering and screening of αCD206-synNotch cells.** (A) Design of the MetBo2-UAS cell line. Initially, a landing pad was established, comprising two selection markers: a G418 resistance cassette, activated by the endogenous ROSA26 promoter upon successful integration, and a nuclear mKate2 cassette. Subsequently, through Φc31 recombinase, this cassette was exchanged for a minCMV-UAS-mCherry cassette also replacing the neomycin resistance cassette for a puromycin one. (B, C) Selected MetBo2-UAS clone exhibited inducible (317.1-fold) mCherry fluorescence upon transfection with Gal4VP64. Scale bar 50 μm. (D) Integration into mROSA26 locus was confirmed by PCR of genomic DNA (2,175 bp). (E) Development of the αCD206-synNotch construct. Four clones of the αCD206-synNotch receptor were isolated and tested for activation with CD206^+^ cells. All clones were analyzed in triplicate except for clone 2. *Green arrows indicate primer binding sites. HA – homology arms. Kan/NeoR – Kanamycin/Neomycin (G418) resistance gene. pA – polyadenylation sequence. NLS – nuclear localization sequence. CAG – Cytomegalvirus immediate enhancer/*β*-actin promoter. Pac – puromycin acetylase (puromycin resistance gene). PGK – phosphoglycerate kinase promoter. scFV – single chain variable fragment. H2B – human histone 2B.*.

We sought to achieve stable genomic integration of the synNotch system in MetBo2 cells, a bone metastasis cell line derived from mouse mammary tumor background.^23^ We chose ROSA26 safe harbor^24^ for the integration of the reporter cassette. The strategy was adapted from Malaguti et al. (Figure 2A).^25^ First, a landing pad was established using CRISPR/Cas9-guided integration through homology-directed repair (HDR). The landing pad consisted of a nuclear mKate2 expression cassette (CAG-mKate2-3xNLS), with an upstream promoterless Neomycin resistance (NeoR) open reading frame (ORF), expressed exclusively upon correct targeting of the construct downstream of the ROSA26 endogenous promoter. The whole landing pad cassette was flanked with the attP50 recombination sites for later Φc31-mediated cassette exchange. Following antibiotic selection and clonal isolation of mKate2^+^ cells, the UAS-mCherry reporter cassette was integrated through Φc31-mediated cassette exchange (RMCE). The reporter cassette consisted of a puromycin resistance (Pac) ORF at the 5′ end of the UAS-mCherry cassette. Following RMCE and antibiotic selection, the mKate2^–^ cells were sorted into single cells. Expanded monoclonal cell populations were tested for activation upon transfection with Gal4VP64 transcriptional activator, and the best performing MetBo2-UAS clone (317.1-fold activation) was chosen for further experiments (Figure 2B, C). Genomic integration into the mROSA26 safe harbor was also validated through PCR on genomic DNA (Figure 2D)

The αCD206 synNotch receptor cassette was integrated in MetBo2-UAS cells through a PiggyBac transposase-based integration (at randomly dispersed locations in the genome). The receptor architecture consisted of a αCD206 nanobody,^22^ fused to the Notch core domain and a Gal4VP64 transcriptional activator (Figure 2E). Downstream from the receptor cassette, we integrated a lineage tracking component, H2B-BFP cassette, which was used as selection marker throughout the first round of FACS sorting. The whole receptor and H2B-BFP construct was flanked by PiggyBac inverted terminal repeats (ITRs). The detailed description of the cell line development methodology is available in the Methods section.

Following co-culture screening of the αCD206 synNotch clone candidates against CD206^+^ sender cells (see the next Results section for the sender cell description), four monoclonal populations of αCD206-synNotch Metbo2 cells were isolated (Figure 2E, Figure 1SA). These clones exhibited different activation levels, as well as varying basal signal levels. Basal activation of the receptor, often referred to as ligand-independent activation (LIA), stems from ligand-independent cleavage of the receptor transmembrane domain and subsequent release of the transcriptional activator.^10,26^ The variability in basal signal levels observed here is likely due to the variability in receptor cassette integration between clones. This is characteristic of random integration systems, such as PiggyBac, and results in different integration event numbers, directly impacting LIA levels. Despite its relatively higher basal signal level, clone number 4 (3.1-fold activation) was chosen as the best-performing clone when tested against CD206^+^ sender cells and will be further referred to as αCD206-synNotch.

### Development of CD206^+^ Sender Cells

**Figure 3:**
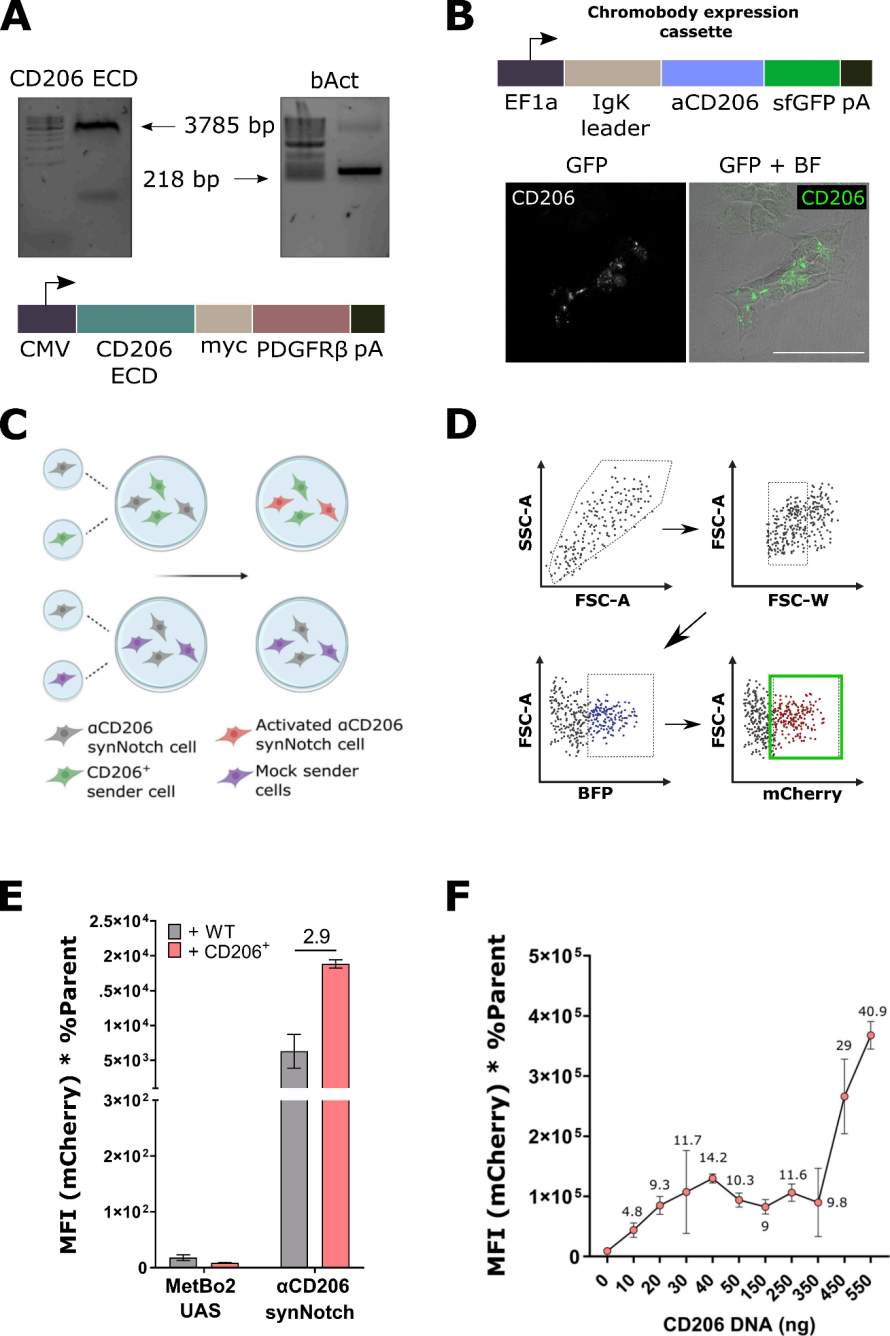
**Development of CD206**^**+**^**sender cells and further characterization of our αCD206-synNotch cell clone.** (A) Extraction of CD206 ECD CDS from cDNA of IL4 stimulated bone marrow-derived mouse macrophages (BMDM). β-actin was used as housekeeping gene for validation of cDNA. (B) Positive staining by αCD206 chromobodies (GFP) of live MetBo2 sender cells transiently expressing the CD206 ECD construct. Scale bar 100 μm. (C) Co-culture strategy for normalization of co-culture conditions among test and control wells. *Created with*BioRender.com. (D) Flow cytometry gating strategy to quantify the mCherry fluorescence of synNotch-positive (BFP^+^) activated cells. *Created with*BioRender.com. (E) αCD206-synNotch clone 4 exhibits a 2.9-fold activation in co-culture with CD206^+^ cells. In comparison, no mCherry signal was observed when using MetBo2-UAS cells as receiver cells, which shows that in synNotch co-cultures the mCherry signal comes solely from receptor activation. (F) αCD206-synNotch demonstrates an increase in activity in response to increasing amounts of ligand transfected in sender cells. Numbers indicate fold increase compared to background activation (synNotch co-cultured with wild-type cells). *CMV – Cytomegalovirus mammalian promoter. HA – Human influenza hemagglutinin tag. Myc – c-myc tag. PDGFR*β – *Platelet-derived growth factor receptor beta transmembrane domain. pA – polyadenylation sequence.*.

We engineered synthetic CD206^+^ sender cells expressing the extracellular domain (ECD) of mouse CD206. The CD206 expression cassette contained an ORF for the CD206 ECD (NM008625.2, 81–3835 nt) fused to the PDGFRβ transmembrane domain (Figure 3A). The CD206 ECD sequence was isolated from the cDNA of BV6 mouse bone-marrow-derived macrophages (BMDMs) following their induction with interleukin-4 (Figure 3A). For this study, synthetic sender cells were preferred over primary macrophages, as this avoided the need for continuous sourcing of large quantities of CD206^+^ macrophages. We chose MetBo2 cells as the synthetic sender cell chassis, so both receptor and sender cells presented the same surface markers, with the exception of CD206 ECD displayed on sender cells. This way, we sought to engineer synNotch cells that reacted solely with CD206 and not with natural ligands on MetBo2 cells. This was crucial for *in vivo* use, so receptor cells would not report (self-)contact with other neighboring cancer cells within the metastatic tumor mass. The CD206 expression cassette was transiently expressed in wild-type MetBo2 cells and validated through immunostaining using αCD206-mNeonGreen chromobodies in an assay developed by Baronaite et al. (Figure 3B)^27^ (See Methods).

### **α**CD206-synNotch Successfully Targeted CD206^+^ Cells *In Vitro*

The co-culture strategy used to determine receptor activation is depicted in Figure 3C. Here, αCD206 synNotch cells were co-cultured with either ligand-presenting sender cells (CD206^+^ cells) or mock sender cells (wild-type MetBo2 cells). This allowed normalization of receptor activation between test and control groups by maintaining equal numbers of receiver-to-sender cells in co-cultures. Additionally, our flow cytometry gating strategy to quantify the percentage of activated cells is depicted in Figure 3D. Here, BFP is associated with constitutive H2B-BFP expression from the receptor cell population, and mCherry is the reporter expressed upon contact with sender cells and a marker of activated cells. The best performing αCD206-synNotch clone from the initial screen was retested for activation and exhibited a 2.9-fold activation when co-cultured with MetBo2-CD206^+^ cells (Figure 3E, Figure 1SB). Basal activation (in the absence of ligand-presenting cells) is observed in αCD206-synNotch but not in MetBo2-UAS cells, confirming that synNotch cell basal activation is dependent on receptor expression and does not result from “leaky” reporter expression. Moreover, the clone demonstrated a dose-dependent activation pattern, with a sharp increase in activation when the sender cells were transfected with more than 450 ng of the ligand expressing vector in a 48-well plate (Figure 3F), suggesting that increased receptor activation can be achieved at higher ligand concentrations.

### **α**CD206 synNotch Exhibits Cross-Specificity with Other Ligands

**Figure 4:**
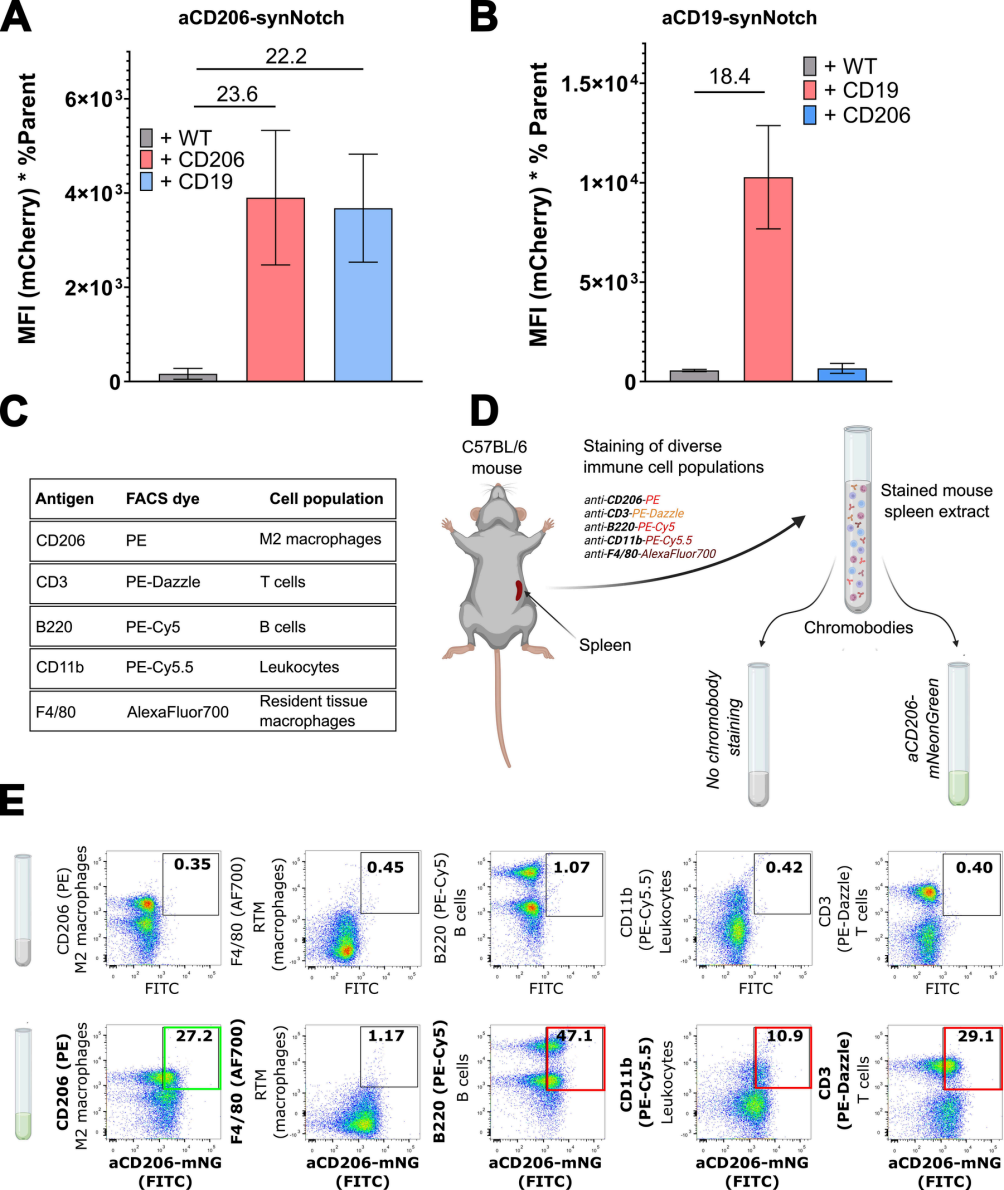
**Testing the specificity of the αCD206 synNotch**. (A) αCD206-synNotch cells exhibited significant cross-reactivity when presented to CD19^+^ cells. (B) αCD19-synNotch cells exhibited no cross-reactivity when co-cultured with CD206^+^ sender cells. (C) The list of antibody antigens and corresponding conjugated dyes used to stain the C57BL/6 mouse spleen extract for specific immune cell subpopulations. (D) The mouse spleen extract was stained with a mix of antibody conjugates in a single-pot reaction. Equal parts of the mix were then stained with αCD206-VHH fused to mNeonGreen (chromobodies). *Created with*BioRender.com. (E) Flow cytometry evaluation of co-staining with the various immune cell subpopulation specific marker dyes: (top) no chromobody co-staining, and (bottom) co-staining with αCD206-mNeonGreen. Numbers indicate the percentage of the co-stained populations.

To better characterize and assess the suitability of αCD206-synNotch in preparation for *in vivo* applications and the targeting of CD206^+^ macrophages, we tested the αCD206-synNotch cells for cross-reactivity with cells overexpressing an irrelevant surface ligand: human CD19, a distinct marker of B cells and a commonly used ligand in other synNotch applications.^10,12,13,19^ We engineered a human CD19^10^ expression vector (Figure S1C) and generated MetBo2-CD19^+^ sender cells (Figure S1D) for co-culture experiments in parallel with CD206 sender cells. Interestingly, αCD206-synNotch exhibited activation (22.2-fold increase) when co-cultured with CD19^+^ sender cells (Figure 4A). Moreover, we observed significant fluctuations in synNotch activity over passages, with αCD206-synNotch activation levels reaching 23.6-fold, compared with the 2.9-fold activation measured previously. The possible reasons for fluctuations in the receptor activation are considered in the Discussion section. For comparison, we tested the αCD19-synNotch^10^ architecture for reciprocal cross-reactivity with MetBo2-CD206^+^ sender cells and did not observe any.

To assess whether synNotch cross-reactivity is due to the low specificity of the small antibody domain used as the synNotch extracellular domain (ECD), we tested the αCD206 VHH for cross-reactivity against various endogenously expressed murine immune cell markers. Through our testing platform, we evaluated the affinity of αCD206-mNeonGreen chromobodies for various immune cell types from a C57BL/6 mouse spleen extract. First, this spleen extract was stained with five conjugated antibodies specific to distinct immune murine cell markers: CD206 (Phycoerythrin, PE), CD3 (PE-Dazzle), B220 (or CD45, PE-Cy5), CD11b (PE-Cy5.5) and F4/80 (AlexaFluor700) (Figure 4C, D). These markers corresponded to five different immune cell populations: CD206^+^ pro-inflammatory macrophages, T cells, B cells, leukocytes and F4/80^+^ resident tissue macrophages, respectively. The dye-stained extract was then cross-stained with αCD206-mNeonGreen chromobodies. The αCD206 VHH reacted with CD206^+^ M2 macrophages as expected but cross-reacted with most other immune cell populations tested (B220^+^ B cells, CD11b^+^ macrophages and CD3^+^ T cells), showing poor specificity for its cognate target and preventing its use as an affinity domain for synNotch.

## Discussion

In recent years the use of synNotch receptors has been widely demonstrated in a variety of applications, both *in vitro* and *in vivo*.^9,10,12−14,16,18,18−21,25^ The majority of such applications are within the field of oncology, where synNotch has been employed to target and eliminate malignant cells. However, to the best of our knowledge, none of these research applications have reported receptor cross-specificity. Here, we report that our newly developed synNotch receptor exhibits cross-specificity with several cell surface markers.

The cross-reactivity of the small antibody domain we used as synNotch ECD was evaluated using a mouse spleen extract as a pool of immune cells presenting various surface markers. Our findings suggest that applying our synNotch system *in vivo* presents significant challenges due to the potential activation of synNotch cells by incorrect interaction partners, resulting in false positives. For instance, while using our αCD206-synNotch to target CD206^+^ macrophages, the receptor is likely to report cell contact with B cells (CD19^+^), which are abundant at both the primary tumor and metastatic sites.^1^ Moreover, the activation of synNotch reporter cells by nontarget immune cells is likely to occur shortly after injection while circulating in the bloodstream and prior to the establishment of primary and secondary tumors in mice. This is a crucial caveat for *in vivo* applications of receptor-based systems, due to the possibility of false-positive detection events, as well as off-target events that may result in significant side effects in cell therapy contexts.^28^ While our αCD206-synNotch was developed primarily to study cell–cell interactions in a mouse model, there are many synNotch- and other synthetic receptor-based tools being developed for human cell therapy applications.^12,14,16−18^ To our knowledge, most published synNotch receptor studies do not report specificity data, and we would like to advocate for a wider adoption (and reporting) of cross-reactivity testing as an essential part of synthetic receptor development pipelines. Additionally, we think of our study as a demonstration of the vital importance of characterizing individual components of these receptor–reporter systems prior to assembling them in cells. In particular, our findings show that the lack of specificity of our receptor is due to the αCD206 affinity domain itself and demonstrate the need for high-specificity nanobodies and single chain variable fragments (scFVs) to improve the reliability and safety of synthetic receptor systems.

Other measurements that could be used to minimize the detection of false-positives and mitigating the effect of receptor cross-reactivity are to (i) evaluate the duration of synNotch activation and (ii) evaluate the ligand exposure time needed to induce the fluorescent response. Knowing the exact amount of time needed for cell–cell interaction to induce a fluorescent response, as well as knowing the exact response duration, would allow for more precise temporal discrimination between false positive activation and ligand-specific activation. Alternatively, resorting to partially immunodeficient mouse strains may help to reduce the amount of nonspecific receptor activation.^29^ However, this approach is limited to certain applications, as these tools are specifically developed to track interactions with immune cells.

Lastly, we observed that αCD206-synNotch exhibited a variable pattern of activation over experiments, with activation-fold fluctuations between 3- and 24-fold. One possible explanation for this is varying ligand availability on sender cells. CD206^+^ sender cells were generated using transient transfection, which can result in varying levels of expression across individual experiments. We assessed ligand surface expression qualitatively only, by chromobody staining (Figure 3B): staining indicated low CD206 presentation levels, probably due to the difficult export of the large CD206 surface display cassette (around 4 Kb). Therefore, variability in cell transfection and ligand surface presentation might explain the varying receptor activation levels we observed, despite using fixed amounts of ligand plasmid DNA, cell numbers and growth conditions throughout co-culture experiments. The impact of ligand availability on receptor activation is further supported by the dose-dependent activation pattern observed (Figure 3F), where an increase in the amount of the transfected CD206 plasmid led to an increase in receptor activation. Consequently, sender cell ligand surface expression should be monitored quantitatively as well as qualitatively to confidently correlate ligand expression and receptor activation. Furthermore, the generation and use of stable ligand-expressing sender cells should be favored to ensure more consistent co-culture conditions.

Another possible explanation for nonreproducible receptor activation over cell passages is fluctuation in the maintenance of PiggyBac cassette integration. Due to the random nature of PiggyBac integration, some cassettes might undergo epigenetic silencing over cell passages.^30^ Although not performed in this study, this line of testing could provide more insight into our αCD206 synNotch cell line performance over time and, potentially, explain the variable receptor activation levels.

In conclusion, our findings suggest that utilizing partially characterized synNotch (or other synthetic receptor) systems *in vivo* presents potential risks related to receptor cross-reactivity. Therefore, such tools and their applications must be properly characterized and validated by incorporating cross-specificity tests into the standardized receptor testing pipelines.

## Supporting Information Available

Tables S1 and S2 listing relevant constructs and primer sequences; Figure S1 detailing flow cytometry dotplot graphs and CD19 chromobody staining. (PDF)
αCD206 nanobody and CD206 ECD nucleic acid and amino acid sequences. (XLSX)
