# Supplementary material for: Engineering an αCD206-synNotch Receptor: Insights into the Development of Novel Synthetic Receptors
Source: ACS Synth Biol. 2024 Nov 18;13(12):3876–84. doi: 10.1021/acssynbio.4c00149 (PMC11669160; doi:10.1021/acssynbio.4c00149)
Supplement: Supplementary file 1 — sb4c00149_si_001.pdf [file sb4c00149_si_001.pdf]

Supplementary Information

Engineering an anti-CD206-synNotch receptor: insights into the development of novel synthetic receptors

Sofija Semeniuk[1,2], Bin-Zhi Qian[3], and Elise Cachat[1,2*]

[1]Centre for Engineering Biology, University of Edinburgh, Edinburgh EH9 3BF, United Kingdom

[2]Institute of Quantitative Biology, Biochemistry and Biotechnology, School of Biological Sciences University of Edinburgh, Edinburgh EH9 3BF, United Kingdom

[3]Fudan University Shanghai Cancer Center; Department of Oncology, Shanghai Medical College, The Human Phenome Institute, Zhangjiang-Fudan International Innovation Center, Fudan University, Shanghai, China

[*]Corresponding author. Email: elise.cachat@ed.ac.uk

Table S1 : Summary of constructs.

Name	Description	Construct	Notes
Addgene #79125	αCD19-synNotch	PGK→IgKleader→myc→αCD19→ synNotch→ Gal4VP64→WPRE	A gift from Wendell Lim[10]
KPL155	Φc31 recombinase	CMV→Φc31→pA	A gift from Sally Lowell[25]
Addgene #183609	ROSA26 landing pad for creation of MetBo2-RMCE	ROSA HA 5'→Splice Acc→ Kan/NeoR→pA←pA←3xNLS-mKate2←CAG←ROSA HA 3'	A gift from Sally Lowell[25]
pHWu1	αCD206- synNotch	PGK→IgKleader→6xHis→ αCD206→synNotch→ Gal4VP64→WPRE	
pSSe3	CD19 ligand for engineering of MetBo2 $CD19^+$ cells	CMV→IgKleader→HA→CD19→ myc→PDGFRβ→pA	
pSSe14	CD206 ligand for engineering of MetBo2 $CD206^+$ cells	CMV→CD206→myc→PDGFRβ→ pA	
pSSe22	αCD19-synNotch	PB ITR 5'→PGK→IgKleader →myc→αCD19→synNotch→ Gal4VP64→pA→PGK→H2B-TagBFP → pA →3' PB ITR	PiggyBac backbone
pSSe24	αCD206-synNotch	PB ITR 5'→PGK→IgKleader →myc→αCD206→synNotch→ Gal4VP64→pA→PGK→H2B-TagBFP→pA →3' PB ITR	PiggyBac backbone
SP59	PiggyBac transposase	CMV→hyPBase→pA	
pSSe40	UAS-mCherry cassette for RMCE	attB53→Pac→pA→pA←mCherry← minCMV←5xGal4-UAS←attB53	
pSSe59	ROSA26 gRNA and Cas9 vector	U6→gRNA→CMV→3xFLAG-Cas9-T2A-GFP→pA	
UBa1006	αCD206 VHH fused to mNeonGreen	EF1α→IgKleader→αCD206-mNeonGreen→pA	Engineered by Ugne Baronaite (Cachat lab)
UBa0003	αCD206 VHH fused to sfGFP	EF1α→IgKleader→αCD206-sfGFP→pA	Engineered by Ugne Baronaite (Cachat lab)
UBa1007	αCD19 scFV fused to mNeonGreen	EF1α→IgKleader→αCD19-mNeonGreen→TCS-6xHis→pA	Engineered by Ugne Baronaite (Cachat lab)

Table S2 : Summary of key primers.

Name	Sequence	Notes
mROSAwtF	GGCGGACTGGCGGGACTA	Wild-type ROSA26 locus fwd primer; Used for confirming integration with gRNA PCR;
PuroR	CTTCCATCTGTTGCTGCG	Specific to puromycin resistance gene; Used as a reverse primer for confirming the integration of UAS-mCherry cassette with gDNA PCR;
mKateR	TACGAAGACGGGGGCGTGC	mKate2 reverse primer; Used for confirming integration with gDNA PCR;
mActB_F	CTGTCCCTGTATGCCTCTG	Murine β-actin primers used as control during extraction of ligands from cDNA;
mActB_R	ATGTCACGCACGATTTTC	
MRC1_F	CCGCCAGTGTGCTGGAATTCGGAAGATCCACTCTGGGCC	CD206 Gibson primers for extraction from cDNA.
MRC_Rfull	ATGAGTTTTTGTTCGTCGACGCCATAGAAAGGAATCCACGC	
Gibson_CD19_F	CCCAGCCGGCCAGATCTCCCGAGGAACCTCTAGTG	CD19 Gibson primers for extraction from cDNA.
Gibson_CD19_R	GATGAGTTTTTGTTCGTCGACCTTCCAGCCACCAG	

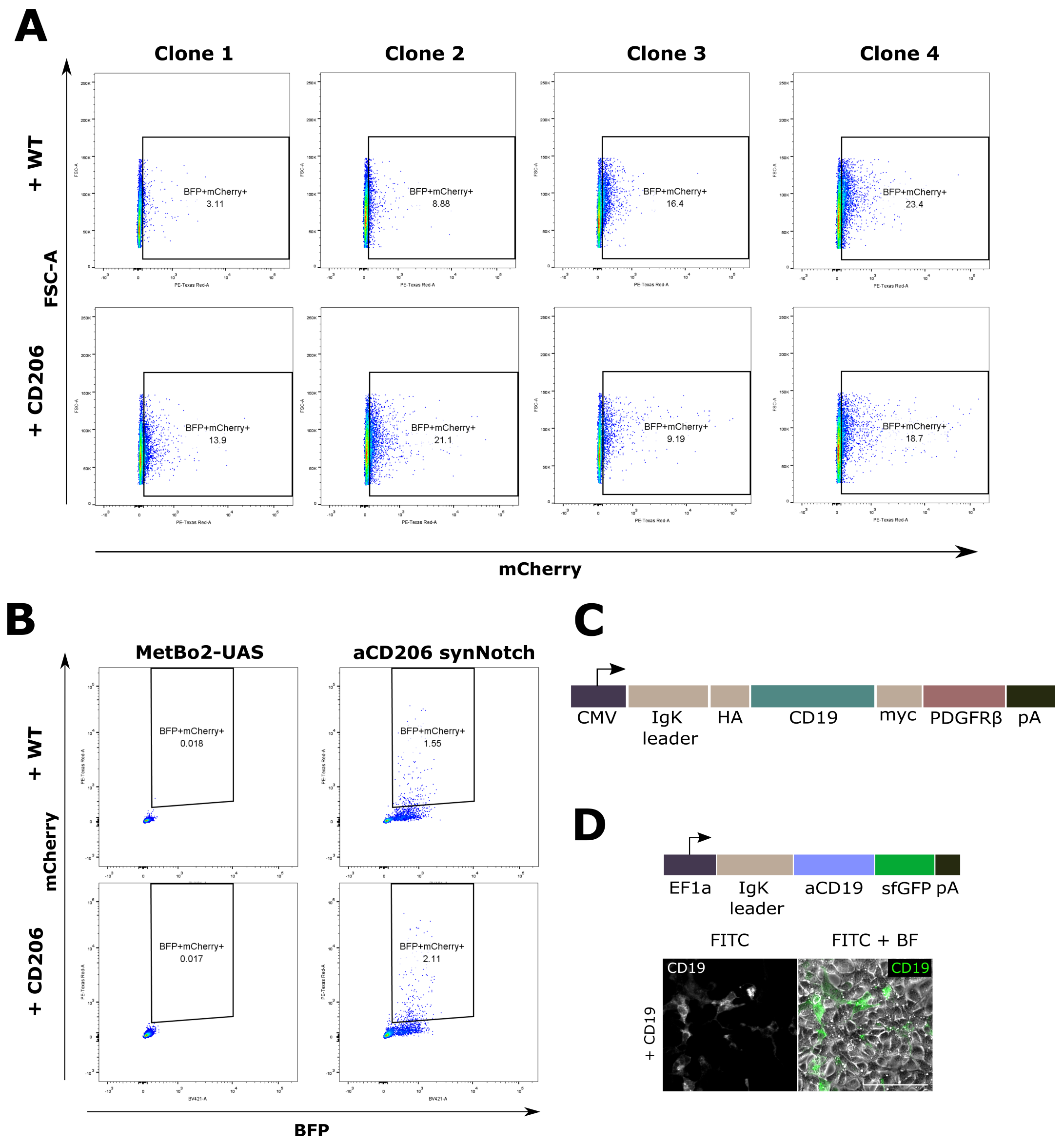

Figure S1 : Development of the αCD206 SynNotch receptor. (A) FACS dot plots representing the distribution of the BFP$^+$mCherry$^+$ (activated synNotch cells) populations. (B) FACS dot plots representing the distribution of the BFP$^+$mCherry$^+$ populations upon repeat testing of the αCD206 synNotch receptor. (C) Structure of the CD19 ligand construct. (D) Chromobody staining of MetBo2 cells, transiently transfected with CD19 surface ligand. Positive staining is indicated by GFP fluorescence. Scale bar 100 μm.
